# Evolving role of Anti-CD20 monoclonal antibodies in rheumatoid arthritis: efficacy, safety, economic perspectives, and future directions—a narrative review

**DOI:** 10.1186/s12916-026-05099-x

**Published:** 2026-08-05

**Authors:** Yang Cao, Xiong-Fei Xu, Ning Ma, Jie Gao, Li-Jiao Cui, Betty Yuen Kwan Law, Vincent Kam Wai Wong

**Affiliations:** 1 Biophysics Laboratory for Innovative Drug Discovery, State Key Laboratory of Mechanism and Quality of Chinese Medicine, School of Pharmacy, Macau University of Science and Technology, Macau, China; 2 Macau University of Science and Technology , Zhuhai MUST Science and Technology Research Institute, Zhuha Guangdong, China

**Keywords:** Rheumatoid arthritis, Anti-CD20 monoclonal antibodies, Rituximab, Biosimilars, Pharmacoeconomics, B-cell depletion, Narrative review

## Abstract

**Background:**

B-cell depletion has established an important therapeutic role in rheumatoid arthritis (RA), particularly in selected patients with an inadequate response to tumor necrosis factor inhibitors or with clinical features favoring rituximab. However, the optimal positioning of anti-CD20 therapy remains constrained by heterogeneous treatment responses, cumulative immunosuppression, retreatment strategies, and differences in treatment accessibility.

**Main text:**

This narrative review synthesizes evidence from randomized controlled trials, long-term extension studies, real-world cohorts, mechanistic investigations, biosimilar studies, and pharmacoeconomic evaluations. Rituximab remains the only anti-CD20 monoclonal antibody with an established clinical role in RA. Newer-generation agents, including ocrelizumab, ofatumumab, and obinutuzumab, incorporate humanized, fully human, or glycoengineered designs that modify antibody-dependent cellular cytotoxicity, complement-dependent cytotoxicity, direct cell death, and immunogenicity. Nevertheless, greater B-cell-depleting potency has not consistently translated into an acceptable long-term benefit–risk profile in RA. This discrepancy is described here as a “next-generation paradox,” reflecting the narrower safety margin required in chronic autoimmune disease than in hematological malignancies. Rituximab biosimilars may improve treatment accessibility, although their economic impact is strongly dependent on regional procurement and reimbursement systems. Biomarker-guided patient selection, ultra-low-dose maintenance regimens, and dual targeting of B cells and BAFF axis represent promising approaches to treatment optimization., but their routine use remains limited by heterogeneous and, in some cases, non-RA-specific evidence.

**Conclusions:**

Rituximab retains clinically relevant value in carefully selected patients with RA, particularly seropositive patients and those for whom alternative targeted therapies are unsuitable. Future research should prioritize RA-specific prospective validation of biomarkers, individualized retreatment strategies, long-term immune safety, and regionally relevant pharmacoeconomic models rather than simply pursuing more potent B-cell depletion.

**Graphical abstract:**

**Figure:**
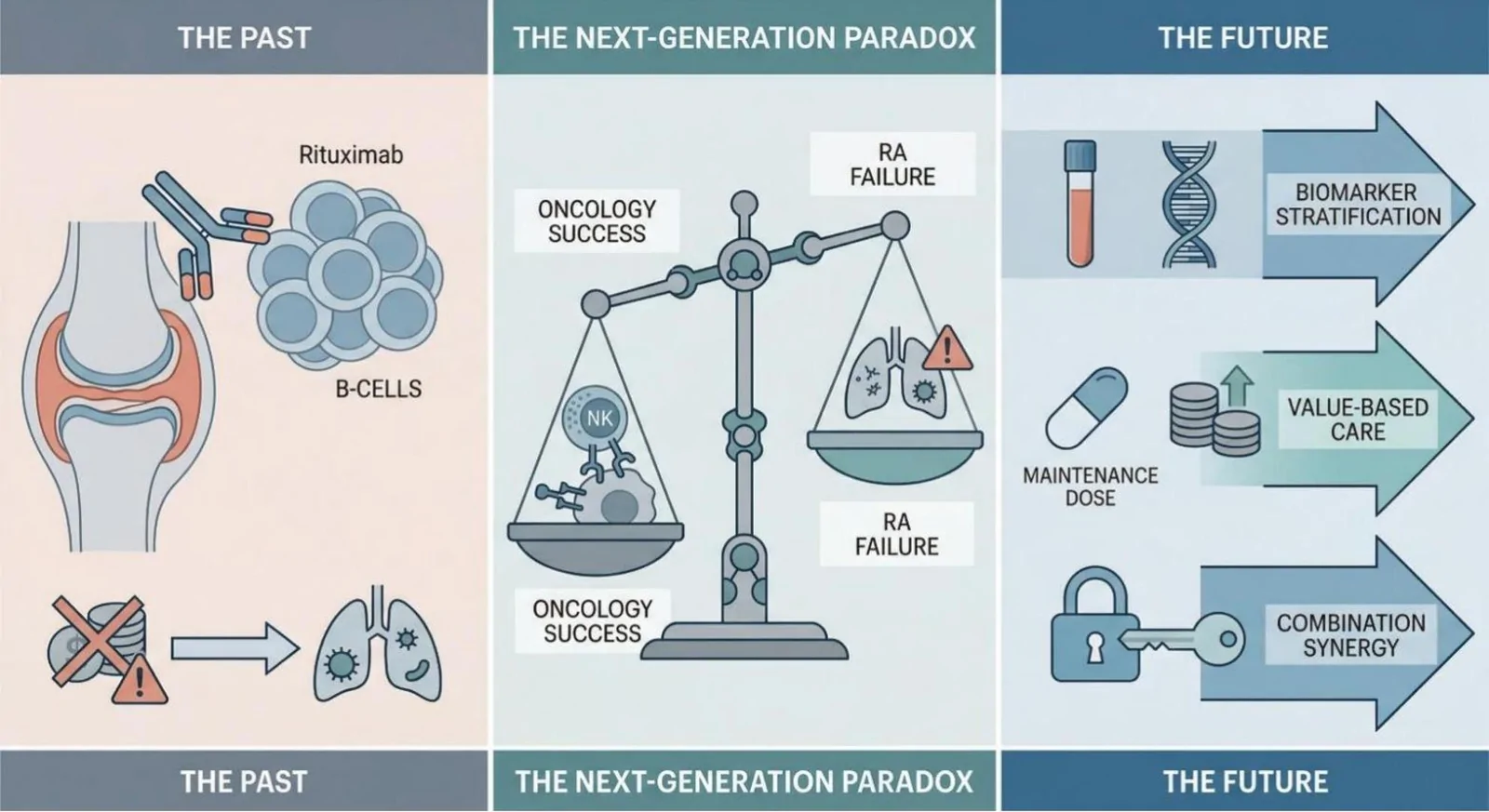

## Highlights

Next-generation paradox: Maximal enhancement of antibody-dependent cellular cytotoxicity (ADCC) may narrow the safety margin of anti-CD20 antibodies in chronic autoimmune diseases.

Economic transformation: Rituximab biosimilars combined with ultra-low-dose maintenance regimens may improve the cost-effectiveness and accessibility of B-cell depletion therapy.

Precision stratification: Synovial and peripheral biomarkers may help identify patients most likely to benefit from targeted B-cell depletion.

Synergistic targeting strategies: Dual targeting of B cells and the the B-cell activating factor (BAFF) pathway may mitigate secondary non-response in selected patients.

Future roadmap: A precision-medicine-oriented framework integrating biosimilars, ultra-low-dose maintenance regimens, and biomarker-based stratification may support individualized long-term RA management.

## Background

Rheumatoid arthritis (RA) is a systemic autoimmune disease characterized by persistent synovial inflammation and progressive structural damage to the joints [1]. Without effective disease-modifying treatment, RA can progress to irreversible dysfunction and increase the risk of all-cause mortality [1]. The global prevalence of RA is approximately 0.5%-1%, which places a substantial economic burden on the medical system, society, and families of patients. According to epidemiological data from the United States, the annual medical expenditure per patient can reach up to $36,053 [2, 3]. In recent years, the clinical application of biological disease-modifying antirheumatic drugs (bDMARDs) has significantly changed the treatment of RA and improved long-term patient outcomes [4]. Among these therapies, anti-CD20 monoclonal antibodies represent an important B-cell-targeted strategy in the treatment of RA [5].

B-cells play an important regulatory role in RA development by producing autoantibodies, presenting antigens, and secreting proinflammatory cytokines [6–9]. CD20 is a transmembrane protein that is continuously expressed from the pre-B cell stage to the memory B-cell stage.However, it is not expressed on terminally differentiated plasma cells or hematopoietic stem cells, making it a suitable therapeutic target for RA [10]. This expression pattern enables CD20-targeted monoclonal antibodies to deplete pathogenic B-cell subgroups while relatively retaining the regenerative ability of B cells and the existing humoral immune function [11]. The development of newer generation anti-CD20 antibodies further emphasizes the optimization of antibody effector function and attempts to improve the efficiency of B-cell depletion [12–14]. Simultaneously, with the continuous application of rituximab and its biosimilars, as well as the continuous exploration of newer-generation anti-CD20 antibodies and combined treatment strategies, treatment options for patients with RA continue to expand. However, clinical drug decision-making has become increasingly complex [15].

Against this background, it is of great significance to re-evaluate the evolutionary effect and clinical positioning of anti-CD20 monoclonal antibodies in the treatment of RA. This article focuses on several key aspects: the structural gaps in the translation of next-generation anti-CD20 antibodies into clinical practice for RA, and the constraints of the “next-generation paradox” on their clinical application; the practical value of rituximab and its biosimilars in improving the accessibility and cost-effectiveness of treatment while maintaining clinical benefits; and the potential contribution of biomarker-guided patient stratification, individualized dosing regimens, and combined intervention strategies targeting the B-cell–BAFF axis to advancing RA management from empirical treatment to precision medicine. Therefore, this article conducts a narrative review of the mechanisms, clinical evidence, transformation challenges, and optimization pathways of anti-CD20 therapy in RA. It also aims to identify current evidence gaps and unmet clinical needs, offering guidance for future research in this field.

## Main text

### Evidence acquisition and narrative synthesis

This article was developed as a narrative review rather than a systematic review or quantitative meta-analysis. A targeted literature search was conducted in PubMed, Embase, and the Cochrane Library to identify English-language publications from January 2000 to April 2026. Relevant regulatory documents, clinical guidelines, pharmacoeconomic reports, and biosimilar-related evidence were also considered where appropriate. The search focused on the mechanisms, clinical efficacy, safety, immunogenicity, biosimilars, pharmacoeconomics, and treatment optimization of anti-CD20 therapy in RA.

Search terms included combinations of “rheumatoid arthritis,” “anti-CD20 antibody,” “B-cell depletion,” “rituximab,” “ocrelizumab,” “ofatumumab,” “obinutuzumab,” “biosimilar,” “B-cell activating factor,” “BAFF,” “belimumab,” “pharmacoeconomics,” “cost-effectiveness,” “biomarker,” and “reduced-dose rituximab.” Priority was given to randomized controlled trials, long-term extension studies, large real-world cohorts studies, clinical guidelines, regulatory documents, pharmacoeconomic studies, and mechanistic studies directly relevant to anti-CD20 therapy.

Evidence was synthesized narratively according to clinical and mechanistic themes. Evidence from hematological malignancies, multiple sclerosis, systemic lupus erythematosus, or other autoimmune diseases was used only to inform mechanistic or translational discussion and was not treated as direct evidence of efficacy in RA. Because this was a narrative review, no Preferred Reporting Items for Systematic Reviews and Meta-Analyses (PRISMA) flow diagram, formal risk-of-bias assessment, or quantitative meta-analysis was performed.

## Multifaceted roles of B Cells in RA pathogenesis

### Autoantibody production

The role of B cells in the pathogenesis of rheumatoid arthritis (RA) is not limited to the secretion of autoantibodies. B-cells can differentiate into plasma cells and produce rheumatoid factor (RF) and anti-citrullinated protein antibodies (ACPAs) [16–18]. These serological abnormalities often appear before clinical symptoms and are associated with pathogenic immune activation and more serious disease progression [19–23].

### Antigen presentation

**Fig. 1:**
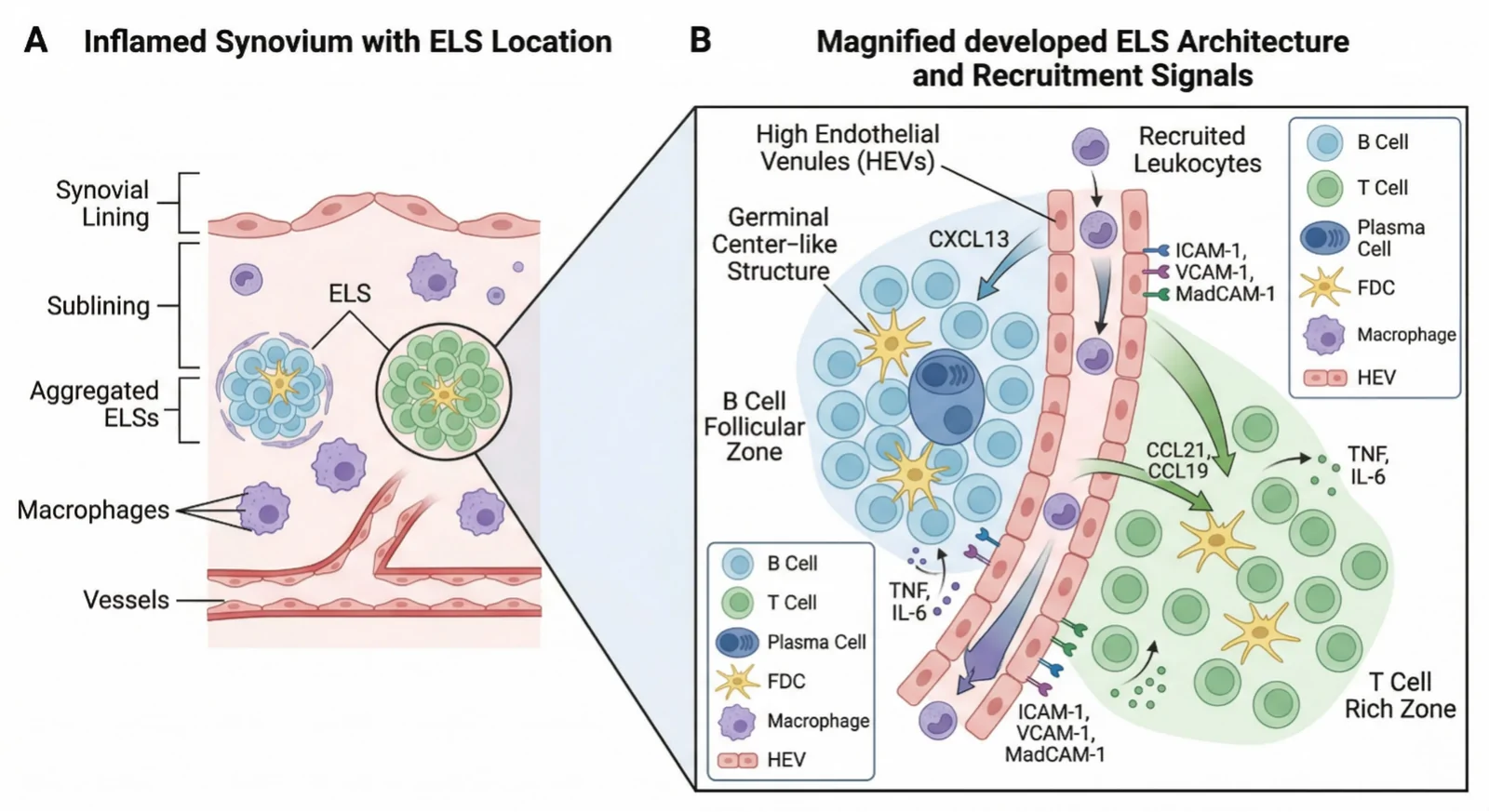
Cellular composition and molecular organization of ectopic lymphoid structures (ELSs) in the RA synovium. (**A**) Schematic representation of inflamed RA synovium showing the spatial localization of aggregated ELSs within the synovial lining. (**B**) Enlarged view of a mature ELS showing functional compartmentalization into B-cell follicle-like areas and adjacent T-cell-enriched zones. The schematic highlights the role of specialized high endothelial venules (HEVs) and chemokine gradients driven by inflammatory mediators such as TNF and IL-6. CXCL13 promotes B-cell recruitment, whereas CCL21 and CCL19 support T-cell homing. This spatial organization may facilitate local antigen presentation and germinal-center-like reactions within the inflamed synovium. *(Note: This original schematic was created by the authors using BioRender.com.*)

As professional antigen-presenting cells (APCs), B-cells can capture citrulline peptide through antigen-specific B-cell receptors (BCRs), internalize them, and present the processed antigen peptides through major histocompatibility complex Class II molecules [24, 25]. Local antigen presentation can activate autoreactive T-cells in the synovial microenvironment [26]. Under the influence of pro-inflammatory chemokines and cytokine gradients, the interaction between pathogenic T-cells and B-cells can promote the formation of ectopic lymphatic structures (ELSs) in the synovial tissue (Fig. 1A) [27]. Mature ELSs may contain follicular-like regions of B cells adjacent to T cell-enriched regions (Fig. 1B). These chemokine gradients maintain the ventricular structure of the spatial region and support the local germinal center-like reaction in inflammatory joints [28]. Therapeutic B-cell depletion may interfere with local antigen presentation in the synovial membrane, thus weakening the activation of pathogenic T cells [29, 30].

### Cytokine secretion

Cytokines derived from B-cells directly shape the microenvironment of synovial inflammation [31]. Pathogenic B-cell subgroups produce TNF-α, IL-6, and lymphotoxins [32, 33]. These mediators play different roles: TNF-α can activate synovial fibroblasts, while IL-6 participates in systemic acute phase reaction [34, 35]. In contrast, regulatory B-cells can inhibit macrophage-mediated inflammatory response by producing IL-10 [36–38]. However, in chronic RA, this regulatory balance is disrupted, and the continuous production of pro-inflammatory cytokines overwhelms the endogenous immunomodulation mechanism [31, 39, 40].

### Interaction with the synovial microenvironment

In recent years, single-cell RNA sequencing (scRNA-seq) technology has changed our understanding of RA synovitis, transforming it from isolated cell abnormalities to intercellular interaction networks with spatial organization [41]. In this network, B-cells interact with peripheral helper T cells (Tph), synovial macrophages, and invasive synovial fibroblasts, and their functional range exceeds that of autoantibodies. These interactions are regulated by chemokine gradients. For example, CXCL13 expression is often increased in the synovium of seropositive patients, promoting local B cell recruitment and the formation of mature ectopic lymphoid structures (ELSs) [42]. ELSs may function as localized immune niches that are relatively protected from systemic immune regulation, supporting in situ affinity maturation of autoantibodies and amplifying synovial inflammation. CD20-directed B-cell depletion may partially remodel this spatial pathology by targeting key B-cell components within the synovium and weakening ELS-associated inflammatory feedback loops [43].

In summary, the multiple functions of B cells in autoantibody production, antigen presentation, cytokine secretion and synovial microenvironment remodeling provide a biological basis for targeting CD20-positive B cells in RA.

## CD20 as a therapeutic target for B-cell-directed therapy

CD20 is a relatively selective therapeutic target for regulating the B cell-driven inflammatory caspase response in RA [44]. As a member of the MS4A transmembrane protein family, CD20 is continuously expressed from the pre-B cell stage to the memory B-cell stage, but not expressed in hematopoietic stem cells and mature plasma cells [45–47]. Therefore, the therapy for CD20 can deplete the specific pathogenic B cell groups without affecting hematopoietic stem cells and plasma cells, which is helpful to maintain the regenerative ability of B-cells and the existing humoral immune function [45–47]. From the physiological mechanisms, CD20 promotes B-cell activation by regulating calcium influx and the B-cell receptor (BCR) signaling pathway [48]. Therefore, anti-CD20 monoclonal antibodies can play a dual role: on the one hand, they can deplete the B-cell groups involved in autoantibody production, antigen presentation, and pro-inflammatory cell secretion; on the other hand, they may weaken the co-stimulation signal required for local T cell activation [49–51]. The B-cell depletion effects of different anti-CD20 antibodies may involve mechanisms such as complement-dependent cytotoxicity, antibody-dependent cell-mediated cytotoxicity, antibody-dependent cell phagocytosis, and direct induction of cell apoptosis. Its relative contribution varies depending on the type of anti-CD20 antibody and local immune microenvironment [52–56]. However, the kinetics of B-cell reconstitution, the optimization of the interval between retreatment and the risk of immunodeficiency associated with long-term repeated treatment remain the main clinical challenges [11, 57–61].

## Rituximab: clinical evidence for B-cell-targeted therapy

### Mechanisms of action

**Fig. 2:**
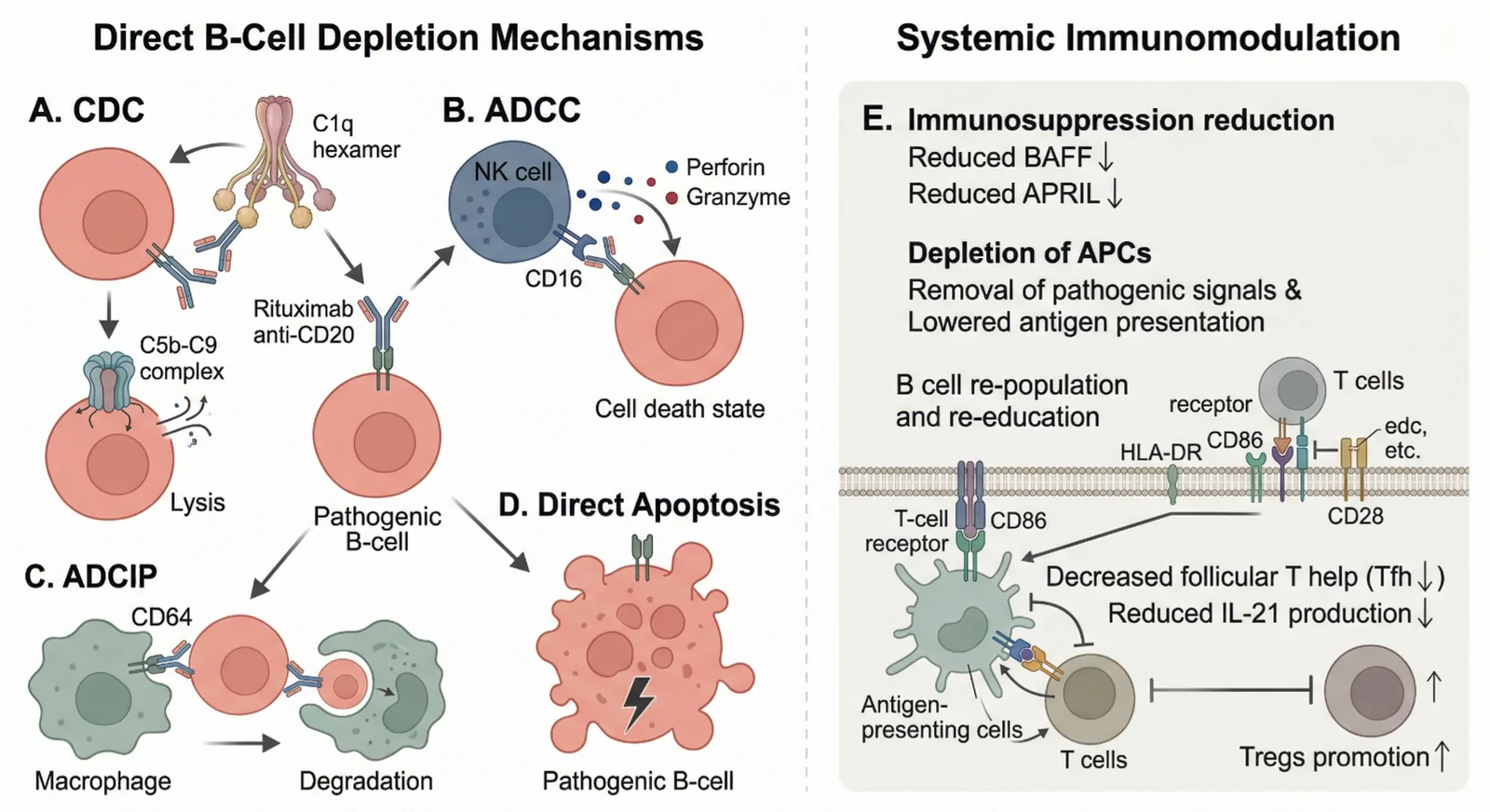
Mechanisms of rituximab in RA: targeted B-cell depletion and subsequent systemic immune regulation. The therapeutic effects of rituximab are mediated by immediate CD20-positive B-cell depletion and subsequent remodeling of immune-cell interactions. (**A**) Complement-dependent cytotoxicity (CDC): After binding to CD20 on the B-cell membrane, rituximab promotes C1q recruitment and activation of the classical complement pathway, leading to C5b–C9 membrane attack complex formation and complement-mediated target-cell lysis. (**B**) Antibody-dependent cellular cytotoxicity (ADCC): Natural killer (NK) cells bind to the Fc region of rituximab through FcγRIIIa/CD16 and release perforin and granzymes, resulting in targeted B-cell death. (**C**) Antibody-dependent cellular phagocytosis (ADCP): Macrophages recognize rituximab-opsonized B cells through Fcγ receptors and mediate phagocytosis followed by lysosomal degradation. (**D**) Direct apoptosis: CD20 cross-linking may induce intracellular death signaling, membrane blebbing, and programmed target-cell death independent of complement or immune effector cells. (**E**) Systemic immunomodulation: B-cell depletion may reduce antigen presentation and co-stimulatory signaling, attenuate pathogenic T-cell activation, modulate macrophage inflammatory activity, and promote partial restoration of regulatory immune pathways, including increased regulatory T-cell activity and production of immunoregulatory cytokines such as TGF-β and IL-10. These downstream effects may contribute to the clinical benefit of rituximab beyond direct B-cell removal. (*Note: This original schematic was created by the authors using BioRender.com.*)

Rituximab mainly induces B-cell depletion through four mechanisms **(**Fig. 2**)**: complement-dependent cytotoxicity (CDC), antibody-dependent cell-mediated cytotoxicity (ADCC), antibody-dependent cell-mediated phagocytosis (ADCP) and direct induction of cell apoptosis (DCD) [62–64]. When rituximab binds to CD20 on the B-cell membrane, it can promote the aggregation of CD20 and activate the downstream effect mechanism. This conformational change can enhance the recruitment of C1q and activate the classical complement cascade reaction, which ultimately leads to the formation of the membrane attack complex (MAC) and complement-mediated cell lysis. Natural killer (NK) cells can also bind to the Fc segment of rituximab through FcγRIIIa, release perforin and granzyme, and mediate ADCC [65, 66]. In addition, macrophages can phagocytize conditioned B-cells through ADCP, and CD20 crosslinking can further promote the apoptosis of target cells [67]. B-cell depletion may lead to the repolarization of the systemic immune system. The absence of activation signal from B cells may decrease the expression of activation markers such as HLA-DR, CD69 and CD40L on CD4 + effector T cells [63]. Macrophages may change to a tissue repair phenotype and reduce the secretion of pro-inflammatory TNF [66]. Regulatory T cells can up-regulate the expression of FOXP3 and increase the production of TGF-β and IL-10, indicating that the immunoregulation function has been partially restored [68–73].

### Clinical efficacy

Critical randomized controlled trials have transformed these mechanisms into clinical evidence. As shown in Table 1, the DANCER, REFLEX, SUNRISE, and IMAGE studies all showed that compared with methotrexate (MTX) monotherapy or placebo controls, rituximab can improve the improve the American College of Rheumatology 20%, 50%, and 70% response rates (ACR20/50/70) and delay radiographic progression [53, 74–76]. These trial data can support the clinical positioning of rituximab as an evidence-based B-cell depletion therapy in selected RA populations. Although rituximab has shown efficacy in MTX-naïve RA patients [76, 77], current RA treatment guidelines primarily position rituximab as a targeted option after inadequate response to tumor necrosis factor inhibitors (TNFis), or in selected patients for whom other targeted therapies are unsuitable [15, 74, 78].

**Table 1:** Key efficacy outcomes from pivotal rituximab trials in RA patients

Trial	Patient Population	Treatment Arms	Primary Endpoint	Key Results
DANCER^[53]^	Active RA despite MTX treatment (*N* = 465;79%(367/465)female, 21%(98/465)male)	1. RTX 500 mg×2 + MTX 2. RTX 1000 mg×2 + MTX 3. Placebo + MTX	ACR20 at 24 weeks	ACR20: 54% (500 mg), 55% (1000 mg), 28% (placebo) *p* < 0.001 for both RTX groups vs. placebo
REFLEX^[74]^	TNFi-IR(*N* = 520;81%(421/520)female,19%(99/520)male; mean disease duration 12.1 ± 8.3 years)	1. RTX 1000 mg×2 + MTX 2. Placebo + MTX	ACR20 at 24 weeks	ACR20: 51% vs. 18% (*p* < 0.0001)
SUNRISE^[75]^	MTX-IR(*N* = 512; 82%(419/512)female, 18%(93/512)male; mean disease duration 7.1 ± 7.2 years)	1. RTX 500 mg×2 + MTX 2. RTX 1000 mg×2 + MTX 3. Placebo + MTX	ACR20 at 24 weeks	ACR20: 55% (500 mg), 54% (1000 mg), 23% (placebo) *p* < 0.0001 for both RTX groups vs. placebo
IMAGE^[76]^	MTX-naïve early RA (*N* = 755; 79%(596/755) female, 21%(159/755) male; mean disease duration < 2 years)	1. RTX 500 mg×2 + MTX 2. RTX 1000 mg×2 + MTX 3. Placebo + MTX	Change in mTSS at 52 weeks	Mean change in mTSS: 0.359 (1000 mg), 0.578 (500 mg), 1.079 (placebo) *p* = 0.0019 for 1000 mg vs. placebo; *p* > 0.05 for 500 mg vs. placebo

RTX: Rituximab; MTX: Methotrexate; TNFi-IR: Tumor Necrosis Factor Inhibitor Inadequate Responder; mTSS: modified Total Sharp Score MTX-IR: Methotrexate Inadequate Responders

ACR20: American College of Rheumatology 20% response criteria

Real-world evidence further supports these findings. In the Belgian MIRA Registry(*N* = 649), rituximab showed sustained therapeutic persistence in a highly refractory RA population, with persistence reaching 66.7% over 3.2 years [79–81]. Owing to the intermittent administration of rituximab, disease activity may show a “sawtooth” pattern between treatment cycles. When disease activity reaches moderate levels (such as Disease Activity Score in 28 joints (DAS28) ≥ 3.2), delaying re-treatment may increase the risk of clinical progression. If DAS28 increased by more than 1.2 points compared with the baseline, it can be used to guide timely re-treatment and improve the success rate of secondary disease control. Real-world prediction models consistently show that serum-positive (RF/ACPA positivity) and fewer prior biologic failures are key predictors of sustained response, supporting the earlier consideration of rituximab in appropriately selected seropositive patients [78].

### Safety profile

The overall safety of rituximab is generally acceptable, but long-term B cell depletion can increase the risk of cumulative immunosuppression. Post-marketing pharmacovigilance data and 11-year global multi-center clinical trial expansion data show that its overall safety remains stable, although some adverse events show cumulative characteristics in repeated treatment cycles **(**Table 2**)** [21, 82–85]. Regarding the risk of malignant tumors, the summary analysis of a large-scale registered studies has not shown a statistically significant increase in the incidence of solid tumors or lymphoma in patients treated with rituximab compared to the general population of RA patients [86]. However, some specific long-term risks still need attention, especially progressive hypogammaglobulinemia, rare opportunistic infections, and latent virus reactivation. These risks highlight the necessity of pre-treatment virological screening and continuous immunological monitoring during long-term treatment.

**Table 2:** Long-term safety data from the rituximab global clinical trial program [79]

Safety Outcome	Overall Rate (95% CI) per 100 patient-years	North America	Europe	Asia-Pacific	Latin America
Serious infections	3.94 (3.26–4.75)	3.78 (2.95–4.82)	3.79 (3.01–4.75)	5.12 (3.83–6.84)*	3.59 (2.47–5.21)
Myocardial infarction	0.39 (0.29–0.52)	0.45 (0.30–0.68)	0.41 (0.28–0.59)	0.22 (0.09–0.52)	0.31 (0.14–0.69)
Malignancies (excluding NMSC)	0.74 (0.60–0.91)	0.86 (0.65–1.14)	0.76 (0.58–0.99)	0.43 (0.21–0.86)	0.65 (0.37–1.13)
Fatal adverse events	0.71 (0.56–0.88)	0.68 (0.49–0.95)	0.75 (0.56–0.99)	0.81 (0.46–1.41)	0.54 (0.28–1.05)

**Annual adverse event reporting trends (2010–2023)**:• Serious infections: Remained stable at ~ 3.9 per 100 patient-years throughout the observation period• Cardiovascular events: Decreased from 0.52 to 0.37 per 100 patient-years• Malignancies: Stable at ~ 0.75 per 100 patient-years• Fatal events: Decreased from 0.84 to 0.67 per 100 patient-years

*Statistically significantly higher rate compared to other regions (*p* < 0.05), potentially related to differences in comorbidity profiles and infection surveillance practices rather than direct ethnic susceptibility

NMSC: Nonmelanoma skin cancer

Note: Data represent analysis of 3,194 patients with 11,962 patient-years of exposure from global clinical trials and registries. Regional variations should be interpreted with consideration of differences in comorbidity profiles, concomitant medications, and healthcare access rather than assumed racial/ethnic susceptibilities

### Immunogenicity

Antidrug antibody (ADA) mediated immunogenicity is an important factor affecting the long-term efficacy of rituximab. In RA patients, the incidence of ADA is usually between 1% and 11%, which to some extent reflects the characteristics of rituximab, which has a mouse-human chimeric structure [87]. Neutralizing ADA can combine with circulating drugs, form immune complexes, reduce serum drug concentration, and contribute to secondary non-response [88–90]. However, differences among ADA detection assays complicate the interpretation of associations between ADA status and clinical outcomes [91, 92]. To reduce immunogenicity and improve treatment persistence, antibody engineering research has gradually turned to humanized or fully humanized anti-CD20 monoclonal antibodies with low immunogenicity [87, 93–97].

## Development status and challenges of newer-generation Anti-CD20 antibodies in RA

**Fig. 3:**
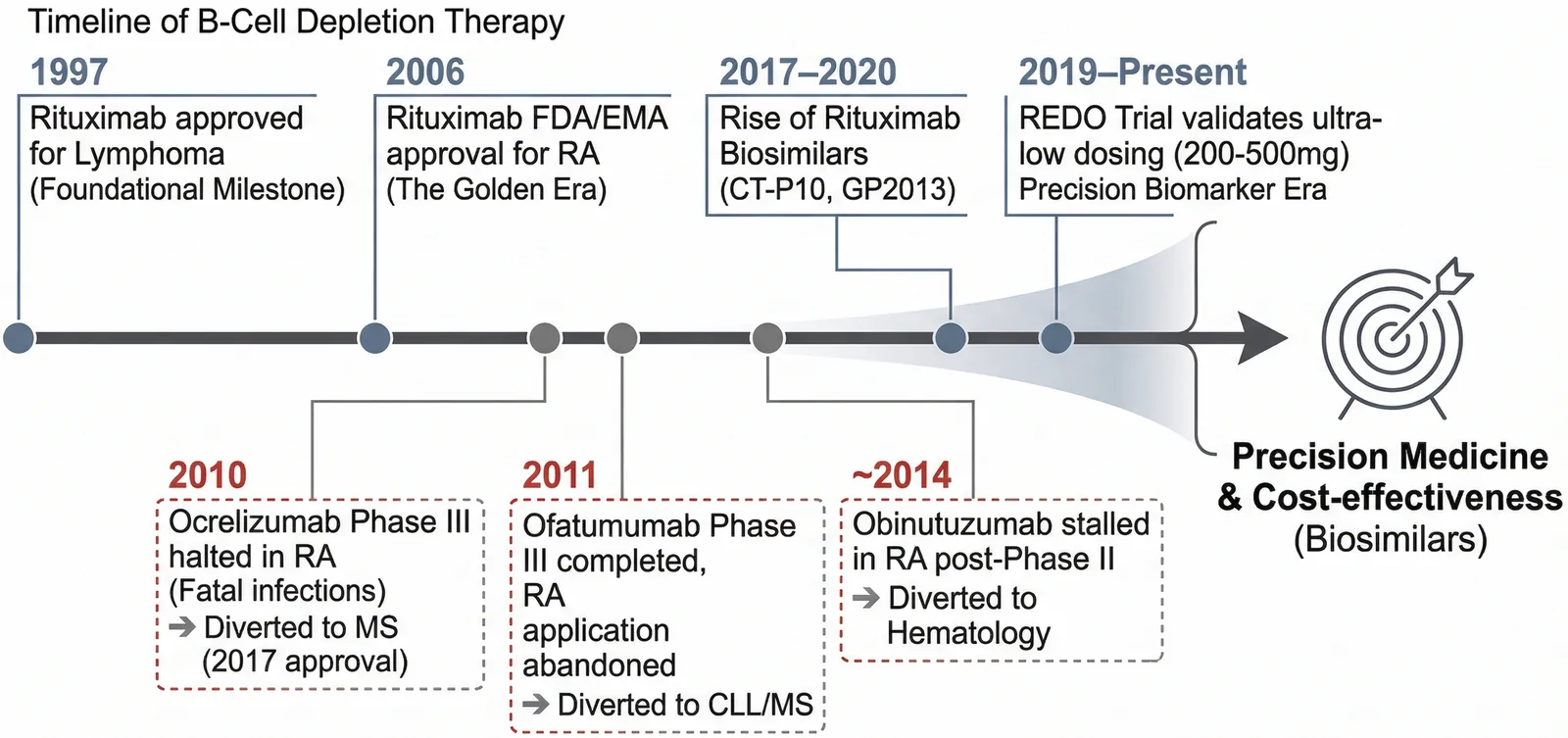
Timeline and development trajectory of anti-CD20 therapies in RA. The development of B-cell depletion therapy in RA has followed a “boom-and-bust” trajectory. Rituximab established the clinical feasibility of CD20-targeted B-cell depletion and was approved for RA after inadequate response to tumor necrosis factor inhibitors. This success stimulated the development of newer-generation anti-CD20 antibodies with humanized, fully human, or glycoengineered structures. However, between approximately 2010 and 2014, the RA development trajectories of several newer anti-CD20 agents became limited or discontinued. Ocrelizumab showed clinical activity but was not pursued further in RA because of unacceptable infectious safety concerns in chronic autoimmune disease. Ofatumumab was redirected toward indications with clearer clinical and commercial positioning, including chronic lymphocytic leukemia and multiple sclerosis. Obinutuzumab, despite enhanced direct cell death and Fc-mediated effector functions in B-cell malignancies, has not established a clear clinical role in RA. In recent years, the field has entered a more pragmatic phase, driven by rituximab biosimilars, cost-containment strategies, ultra-low-dose maintenance regimens, and biomarker-guided patient selection. This trajectory highlights the need to balance antibody potency, long-term safety, clinical positioning, and pharmacoeconomic feasibility in RA. (*Note: This original schematic was created by the authors using BioRender.com.*)

To address the clinical limitations of rituximab, particularly the immunogenicity and infusion-related reactions, researchers have developed the newer-generation anti-CD20 monoclonal antibodies via structural optimization. However, although their translational trajectories in RA have been uneven (Fig. 3). Using rituximab as a benchmark, Table 3 summarizes the key characteristics of ocrelizumab, ofatumumab, and obinutuzumab in terms of molecular structure, clinical properties, and transformation applications. The results show that these three antibodies adopt unique molecular modification strategies: reducing immunogenicity by minimizing the proportion of mouse proteins in the antibodies and regulating the effective functional activity range of the antibodies. These molecular differences may partly explain their different clinical efficacy, safety characteristics, and transformational application results in RA, multiple sclerosis, and hematological malignancies.

**Table 3:** Comparison of rituximab and next-generation anti-CD20 antibodies

Feature	Rituximab	Ocrelizumab	Ofatumumab	Obinutuzumab
Structure	Chimeric	Humanized	Fully human	Humanized, glycoengineered
CD20 Epitope	Type I	Type I	Type I (unique)	Type II
ADCC	+	++	++	+++
CDC	+++	+	+++	+
Direct Cell Death	+	+	+	++
FDA Approval Date (RA)	2006	Not approved for RA*	Not approved for RA**	Not approved for RA
EMA Approval Date (RA)	2006	Not approved for RA*	Not approved for RA**	Not approved for RA
NMPA Approval Date (China)	Not approved for RA	Not approved for RA	Not approved for RA	Not approved for RA
Treatment Duration	6 months	6 months	Variable	Under investigation
Dosing Frequency	Biannual	Biannual	Monthly to quarterly	Under investigation
Immunogenicity	Moderate	Low	Very low	Low
Cost†	$$	$$$	$$$	$$$
Main Side Effects	Infusion reactions, infections	Infusion reactions, infections	Infusion reactions, infections	Infusion reactions, neutropenia

*Ocrelizumab was investigated for RA, but development was discontinued in 2010; approved for multiple sclerosis in 2017

**Ofatumumab was studied for RA but not pursued for approval; approved for chronic lymphocytic leukemia (2009) and multiple sclerosis (2020)

†Relative cost: $ = lower cost; $ = higher cost

ADCC: Antibody-Dependent Cellular Cytotoxicity; CDC: Complement-Dependent Cytotoxicity; FDA: Food and Drug Administration; EMA: European Medicines Agency; NMPA: National Medical Products Administration

### Ocrelizumab

Ocrelizumab is a humanized anti-CD20 monoclonal antibody, and its binding epitope is highly overlapped with rituximab. Compared with rituximab, ocrelizumab has a slower antigen-antibody dissociation rate, stronger ADCC activity, and lower CDC activity [30, 98]. Phase III STAGE trials showed clinical efficacy in RA patients with inadequate response to methotrexate (MTX), with significantly improved ACR20/50/70 response rates compared with placebo [99]. However, its RA development project has been terminated because the risk of severe opportunistic infection exceeded the acceptable safety threshold for the treatment of chronic autoimmune diseases [100]. In contrast, ocrelizumab has successfully achieved clinical transformation in the field of neurology and it has become an approved important disease-modifying treatment drug for multiple sclerosis (including primary progressive multiple sclerosis) [101, 102].

### Ofatumumab

Ofatumumab is a fully humanized type I anti-CD20 monoclonal antibody, which can bind to the membrane-proximal epitope involving the small and large extracellular loops of CD20. This binding topology can enhance the activity of CDC and achieve a strong B cell-killing effect, even in cell population with relatively low CD20 expression levels [103, 104]. Its fully humanized structural characteristics is associated with low immunogenicity and stable drug exposure [96]. However, the RA treatment landscape has formed a highly competitive pattern, especially with the advent of subcutaneously injectable biological agents and orally targeted synthetic DMARDs. Therefore, the clinical development of the drug in the treatment of rheumatism has not been pursued further. In contrast, the focus of research and development has shifted to indications with more obvious competitive advantages, including chronic lymphocytic leukemia (CLL) and relapsing-remitting multiple sclerosis (MS) whose subcutaneous dosage forms show unique clinical application value and commercial potential [105–108].

### Obinutuzumab

**Fig. 4:**
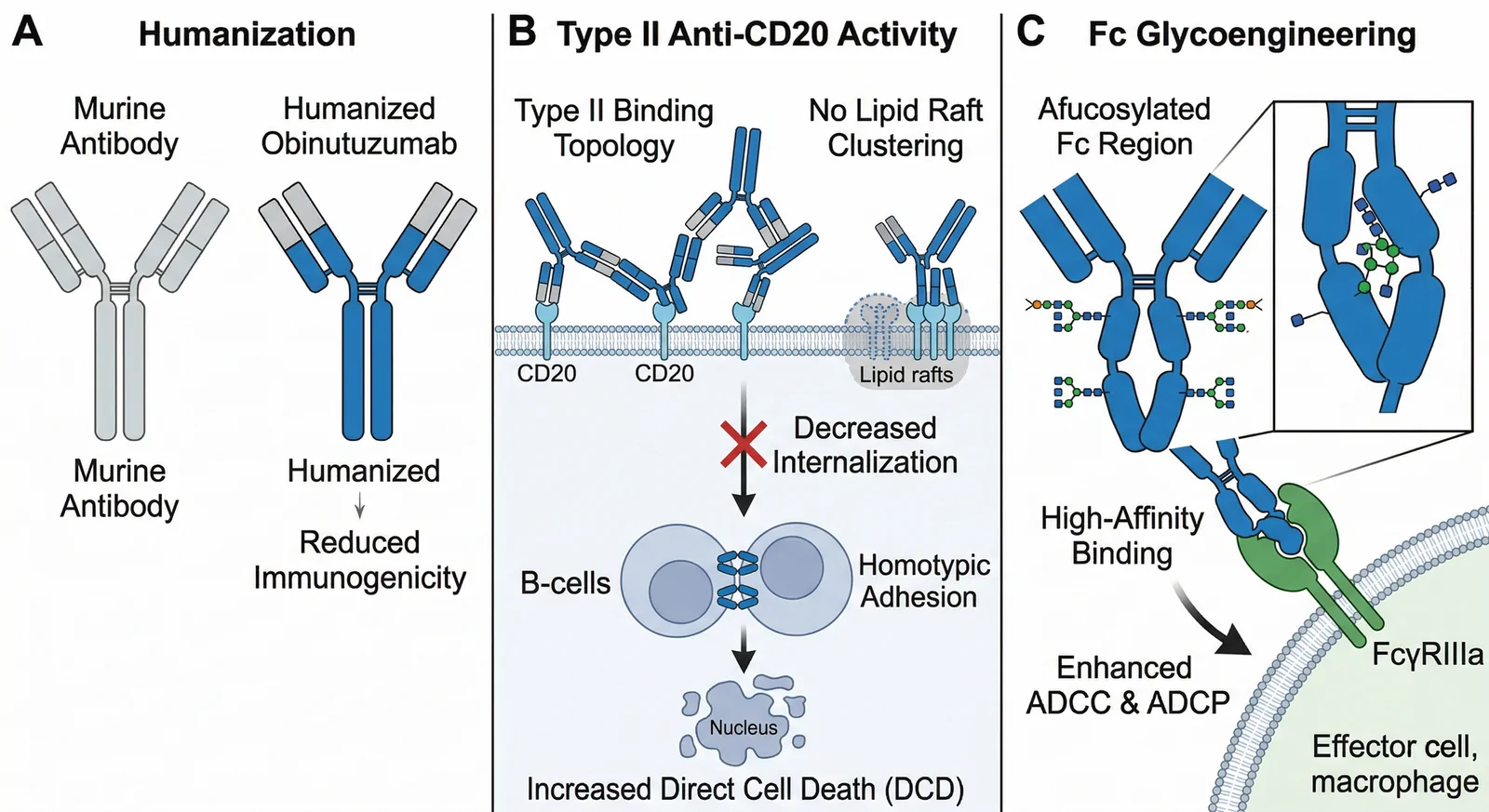
Structural and mechanistic optimization of the type II anti-CD20 antibody obinutuzumab. Obinutuzumab illustrates how antibody engineering can alter CD20-binding behavior and Fc-mediated effector function. (**A**) Humanized and glycoengineered design: Obinutuzumab is a humanized type II anti-CD20 monoclonal antibody with glycoengineered Fc modifications. Its humanized structure reduces murine antibody components, while Fc glycoengineering is designed to enhance interaction with innate immune effector cells. (**B**) Type II CD20-binding topology: Unlike type I antibodies such as rituximab, obinutuzumab does not efficiently redistribute CD20 into lipid rafts. This binding topology reduces rapid internalization and degradation of the antibody–target complex and promotes homotypic B-cell adhesion and direct cell death (DCD). (**C**) Fc defucosylation and enhanced effector function: Fc defucosylation increases affinity for FcγRIIIa on natural killer cells and other innate immune effector cells, thereby enhancing antibody-dependent cellular cytotoxicity (ADCC) and antibody-dependent cellular phagocytosis (ADCP). These properties help explain the strong activity of obinutuzumab in B-cell malignancies, although its clinical translation into RA remains limited by the long-term safety requirements of chronic autoimmune disease. (*Note: This original schematic was created by the authors using BioRender.com.*)

Obinutuzumab is a type II anti-CD20 monoclonal antibody, and its Fc segment is modified by glycoengineered (Fig. 4A). Unlike type I antibodies, obinutuzumab cannot effectively redistribute CD20 into the lipid rafts, thus inhibiting the rapid internalization and degradation of the antibody-target complex [109]. This binding topology can promote the adhesion of homotype B-cells and induce strong direct cell death (DCD), which is an important mechanism that distinguishes it from type I anti-CD20 antibodies (Fig. 4B) [110]. In addition, deglycosylation of the Fc segment can enhance the affinity for FcγRIIIa, thereby enhancing ADCC and ADCP activity and improving target cell depletion efficiency (Fig. 4C) [111, 112]. These characteristics support their therapeutic effects in B-cell malignancies. However, its application in RA remains relatively limited [118]. Theoretically, obinutuzumab may achieve more efficient B cell depletion under conditions of limited effector-cell availability [112]. However, for chronic autoimmune diseases, such as RA that need long-term treatment, the possible benefits of deeper B-cell depletion must be balanced against the risks of long-term immunosuppression and adverse events [113–116].

### Critical appraisal: why the next-generation anti-CD20 therapies stalled in RA

The success of newer-generation anti-CD20 monoclonal antibodies in hematologic malignancies and multiple sclerosis contrasts sharply with the limited development and clinical transformation in RA.This difference can be explained by four interrelated factors.

First, there is a fundamental difference in the risk-benefit threshold between autoimmune diseases and malignancies. For hematologic malignancies, infection risk from potent immunosuppression may be an acceptable trade-off for survival benefit. In RA, by contrast, the therapeutic goal is long-term disease control, functional preservation, and acceptable chronic safety. Although the enhanced ADCC activity of ocrelizumab may improve B-cell depletion efficiency, it triggered an unacceptable infectious toxicity in RA trials, leading to termination of its RA development program [99].

Second, RA synovial tissue in an inflammatory state may form a partially protected local immune microenvironment. Ecliptic lymphatic structures (ELSs) within synovium can support the persistence of pathogenic B-cells in patients with RA. To achieve sufficient B-cell depletion in deep synovial tissue, higher systemic drug exposure may be required, which could amplify peripheral toxicity. This tissue-barrier problem may partly explain why more effective anti-CD20 treatment regimens, such as obinutuzumab, have made limited the development in the field of rheumatology [117].

Third, the heterogeneity of RA limits the applicable population of extensive B-cell depletion strategies. A substantial proportion of patients are serum-negative and may have less B-cell-dominant disease biological characteristics, which reduces the possibility of comprehensive B cell depletion therapy producing consistent efficacy in all RA subgroups.

Fourth, the saturated RA treatment landscape reduces the commercial feasibility of newer anti-CD20 antibodies. Without clear superiority over established biologic DMARDs or rapidly acting oral JAK inhibitors, and given the inconvenience of intravenous infusion, newer-generation anti-CD20 antibodies remain commercially marginalized in RA [78].

The market data further supports this judgement and provides more concrete evidence for the commercial barriers faced by newer anti-CD20 antibodies in RA. According to the IQVIA MIDAS Disease analysis reported, adalimumab remains the dominant drug in the RA market, with a compound annual growth rate of 11% from 2017 to 2022 and a year-on-year growth rate of 13% from 2021 to 2022. At the same time, upadacitinib showed a rapid growth trend in the RA market, with a year-on-year growth rate of 43%, surpassing adalimumab to become an important driver of the absolute growth of the RA market [118]. Prescription path data also showed that TNF inhibitors still dominated the initial targeted treatment options of chronic rheumatoid arthritis after the failure of conventional synthetic disease-modifying antirheumatic drugs (csDMARDs) treatment: in a United States Adelphi Disease Specific Programme analysis, TNF inhibitors accounted for 84.8% (535/631), the not-TNF biologic DMARDs and tofacitinib accounted for only 8.4% and 6.8%, respectively; Among the patients who switched to the second targeted therapy, 60.7% switched to other TNF inhibitors [119]. Similar trends are also seen in Europe and Japan: TNF inhibitors account for 80.8% (1406/1741) and 64.6% (95/147) in EU5 and Japan, respectively [119, 120]. After the failure of first-line targeted therapy, 52.1% of patients in EU5 received second-line targeted therapy, and 54.5% of patients in Japan switched to the mechanism of action [120].

These data show that the treatment pattern of RA still mainly relies on mature TNF inhibition therapy, and the rapid absorption of oral targeted drugs such as JAK inhibition, thus compressing the clinical and commercial space of new intravenous anti-CD20 antibodies. Unless it can show clear efficacy, safety or economic advantages in the screening population of specific biomarkers, its development momentum in RA will still be limited.

In view of the above situation, the research and development focus of the anti-CD20 therapeutic platform has gradually shifted to positioning clearer indications, including multiple sclerosis and hematological malignancies. In these areas, B-cell depletion has become an important therapeutic strategy for specific scenarios [121]. For RA, rituximab may still be one of the most accessible anti-CD20 treatment options in clinical practice for a certain period of time. However, with the popularity of biosimilars, the cost of original rituximab may still limit its accessibility in some medical systems, highlighting the practical importance of rituximab biosimilars in improving the accessibility of treatment.

## Pharmacoeconomic considerations of rituximab biosimilars and Anti-CD20 therapy

Although the pharmacological characteristics of rituximab and newer-generation anti-CD20 monoclonal antibodies shape their therapeutic potential, real-world use is deeply influenced by economic feasibility, payment policy and treatment accessibility. Pharmacoeconomic evaluation is therefore essential and can be comprehensively considered across three domains: clinical evidence, economic impact, and cost-effectiveness.

### Clinical evidence for rituximab biosimilars

The introduction of rituximab biosimilars has significantly improved the accessibility of B-cell-targeted therapy and may reduce the economic burden associated with treatment [122]. Various rituximab biosimilars have been clinically evaluated in head-to-head studies against the original drugs. Large-scale phase III randomized non-inferiority trials, including studies of CT-P10 (Truxima), GP2013 (Rixathon), PF-05280586 (Ruxience), and ABP 798, collectively enrolled more than 1,300 RA patients. The results indicated that its pharmacokinetics, immunogenicity, safety, and ACR20 response rates at 24 and 52 weeks were comparable to those of the original drugs [123–126]. These data support the conclusion that rituximab biosimilars are comparable in terms of efficacy and safety, although regulatory definitions of interchangeability vary across regions [127]. Currently, rituximab biosimilars serve as a crucial tool of enhancing the accessibility and cost-effectiveness of RA treatment, offering viable solutions for clinical practice with constrained resources.

### Economic impact of rituximab biosimilars

The launch of rituximab biosimilars has had substantial economic effects in the treatment of RA. Public drug-procurement data indicate marked regional variation in rituximab biosimilar price reductions (Table 4). In Europe, biosimilars uptake have exceeded 70% in some markets, supported by national policy incentives and centralized tendering mechanisms. In the United States, uptake has been slower, partly because of complex insurance rebate systems, pharmacy benefit manager (PBM) structures, and different regulatory requirements for interchangeability [128]. The decline in the price of biosimilars has brought indirect clinical benefits: the reduction of out-of-pocket costs for patients improves the long-term medication compliance of RA patients, which is expected to improve the treatment sustainability and promote a virtuous cycle between affordability, medication compliance, and long-term disease control [129].

**Table 4:** Approximate publicly listed prices of rituximab and selected biosimilars in selected countries (price per 500 mg vial in USD, as of 2023)

Countries	Reference rituximab	Truxima (CT-P10) / Hanlikang (HLX01)	Rixathon (GP2013)	Ruxience (PF-05280586)
USA	3,500	3,150	3,200	3,050
UK	1,600	1,200	1,150	1,180
Germany	1,800	1,400	1,350	1,380
France	1,700	1,300	1,250	1,280
China Mainland	2,100	~ 550 (NRDL negotiated price)	1,250	Not available
Japan	2,800	2,300	2,250	2,280

Prices are approximate values derived from publicly available formularies, procurement records, or national pricing databases as of 2023 and are presented for contextual comparison only. They should not be interpreted as transaction prices, reimbursement amounts, or RA-specific market-share data. For Mainland China, the originator rituximab product has not been approved for RA; therefore, rituximab-related pricing should be interpreted in the context of local indications, reimbursement policies, and biosimilar availability. However, Hanlikang (HLX01), an independently developed rituximab biosimilar drug, was officially approved by the NMPA for RA in March 2022 and subsequently included in the National Reimbursement Drug List (NRDL) in January 2023, providing a paradigm for biosimilar-driven market access

### Cost-effectiveness analysis of anti-CD20 therapy in RA

After establishing the clinical comparability of rituximab biosimilars and recognizing their market-penetration and price-compression effects across regions, a clinically relevant question emerges: can rituximab or its biosimilars provide sufficient cost-effectiveness advantages to support their use within the broader biologic treatment sequence for RA? Pharmacoeconomic analyses suggest that rituximab, particularly biosimilars, may offer a favorable cost-effectiveness profile in RA patients with inadequate response to TNFis compared with some alternative biologic agents [130, 131]. The ORBIT trial showed that rituximab had clinical efficacy comparable to that of TNFis when used as a first-line biologic agent, while saving £2,118 in healthcare costs per patient per year [132]. Future pharmacoeconomic models should further incorporate individualized RA treatment parameters and evaluate how biomarker-guided selection and ultra-low-dose maintenance regimens may jointly optimize resource allocation and long-term cost-effectiveness [133–135].

## Current challenges and optimization strategies

Although biosimilars have improved the economic accessibility of rituximab, the benefits of B-cell depletion therapy in the real world of RA are still limited by heterogeneity in efficacy, long-term safety, and practice. The following is discussed from the two dimensions of challenge and optimization strategy.

### Current clinical and real-world challenges

#### Primary and secondary non-response

The non-response mode of anti-CD20 therapy can be broadly categorized as primary non-response (defined as insufficient response to the initial treatment plan) and secondary non-response (defined as weakening or disappearance of efficacy after the initial clinical response) [136]. These two models may have different biological mechanisms. The initial non-response may reflect that the drug did not fully penetrate into the inflammatory synovial tissue, or that B-cells accounted for a relatively low proportion in the disease phenotype [137]. Secondary non-response can affect a considerable proportion of initial responders, and the reasons may include ADA-mediated immunogenicity, accelerated drug depletion, decreased serum drug concentration, or early re-proliferation of memory B-cells [138, 139]. These mechanisms suggest that different non-response patterns may require different clinical treatment strategies: patients with primary non-response may need to switch to different treatment regimens with different mechanisms, such as IL-6 inhibitors or JAK inhibitors [137], Patients with secondary non-response mediated by ADA can benefit from the optimization of methotrexate (MTX) regimen, or consider using B-cell targeted therapy with low immunogenicity in certain circumstances [138].

#### Safety management during long-term treatment

Long-term rituximab therapy requires routine immunological monitoring [140]. Currently, the standardized immunoglobulin monitoring remains is incompletely defined, but continuous B-cell depletion may lead to secondary hypogammaglobulinemia. For patients with recurrent severe infection and clinically significant hypogammaglobulinemia, intravenous immunoglobulin (IVIG) alternative treatment can be considered; however, the best timing, administration remains, and cost-effectiveness still need to be further studied [141]. Clinicians should also distinguish between absolute and relative risks: Progressive multifocal leukoencephalopathy (PML) is a serious complication, but it is extremely rare in patients with RA [142, 143]. In contrast, rituximab increases the risk of hepatitis B virus (HBV) reactivation compared to tumor necrosis factor inhibitors (TNFis), indicating the need for strict pre-treatment HBV screening and long-term monitoring [144, 145]

#### Barriers to clinical integration of rituximab biosimilars

Although regulatory and clinical evidence support the use of biosimilars, several barriers remain in real-world implementation [146]. There are significant differences in regulations and implementation standards for the interchangeability of biosimilars across different regions. The U.S.Food and Drug Administration (FDA) has formulated specific substitution standards, whereas the European Medicines Agency (EMA) delegates many substitution decisions to member states [147]. In clinical practice, some doctors may be cautious because they are concerned about cross-immunogenicity or secondary immune response after switching treatment [148]. Patient acceptance is also an important obstacle; negative expectations of “cheaper alternatives” may trigger nocebo effect, leading to aggravation of perceived symptoms and reduced treatment compliance [148]. To overcome these obstacles, it is necessary to establish a structured common decision-making mechanism, strengthen the education of biosimilars on immunogenicity and interchangeability by medical professionals, and improve the post-marketing pharmacovigilance system [149].

### Precise optimization strategies

To address these barriers, research and clinical implementation have increasingly focused on biomarker-guided patient selection, combination strategies, and individualized dosing.

#### Biomarker-guided patient selection

Synovial molecular profiling may help shift RA treatment from empirical drug selection to more precise therapeutic stratification. The R4RA trial showed that rituximab responses were lower in patients whose synovial tissue lacked B-cell-related transcriptomic signatures [150]. Peripheral blood biomarkers may provide more accessible tools for clinical stratification. High baseline RF and ACPA titers have been associated with sustained clinical benefit from anti-CD20 therapy [151]. Similarly, higher baseline peripheral CD20 + B-cell counts may predict a higher likelihood of ACR50 response [152]. In the future, by standardizing the operation process of synovial biopsy procedure and using machine learning methods to integrate the synovial transcriptome, peripheral immune phenotype, and clinical variables, the patient selection regimens for B-cell-targeted therapy can be further optimized [153]

#### Novel combination strategies

**Fig. 5:**
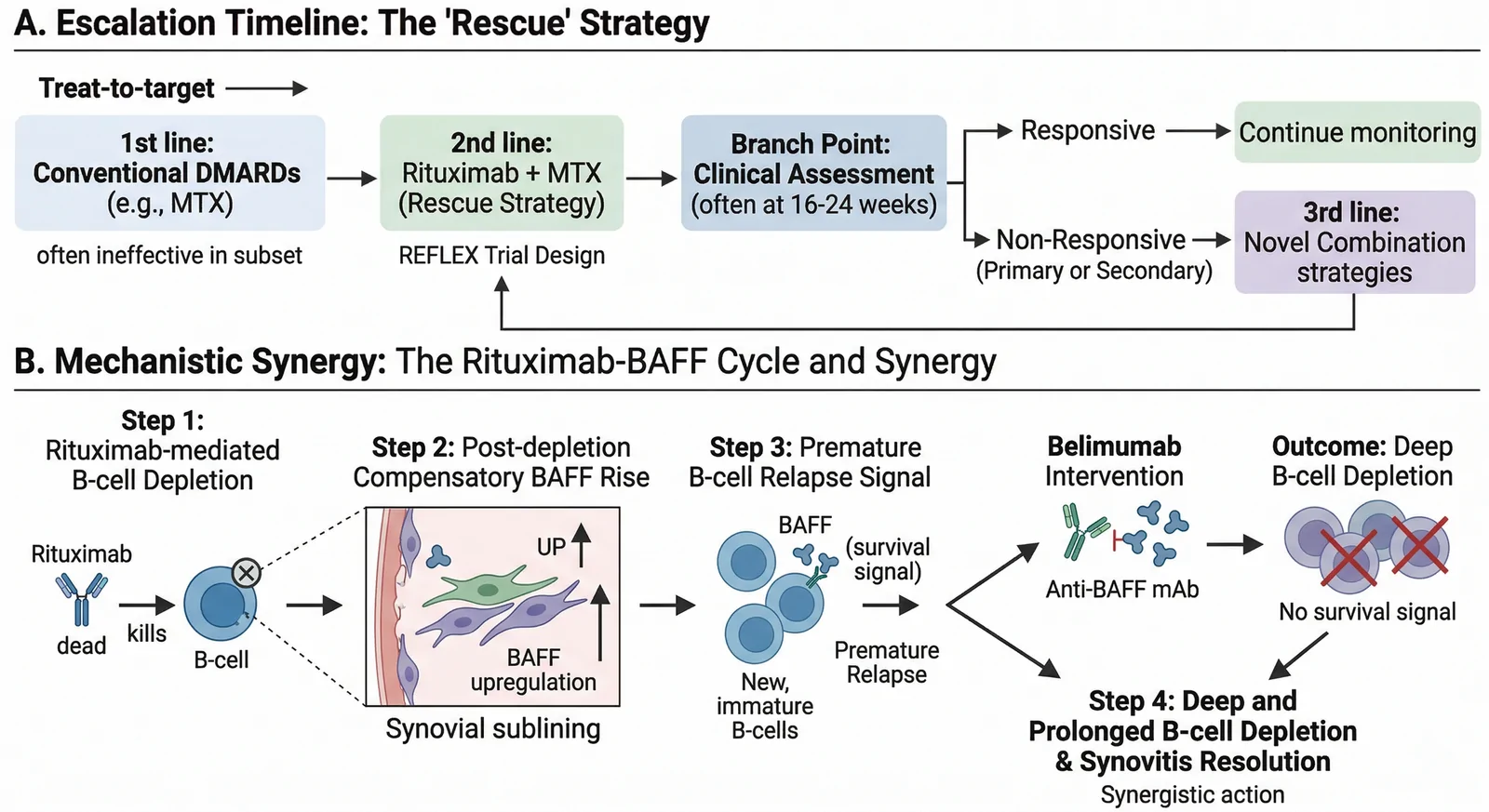
Evolution from empirical sequential switching to rational rituximab-based combination strategies in RA. (**A**) Therapeutic escalation after inadequate response to conventional and biologic DMARDs. The schematic illustrates a treat-to-target escalation pathway beginning with conventional synthetic DMARDs such as methotrexate (MTX), followed by biologic or targeted synthetic DMARDs in patients with insufficient response. Rituximab plus MTX remains an evidence-based option, particularly in selected patients with inadequate response to TNF inhibitors or in those for whom alternative targeted therapies are unsuitable. Patients with primary non-response or secondary loss of response after B-cell depletion may require mechanistically rational alternative or combination approaches. (**B**) Mechanistic rationale for B-cell–BAFF axis dual targeting. The B-cell–BAFF axis dual-targeting strategy refers to a therapeutic approach that combines CD20-mediated B-cell depletion with blockade of BAFF-dependent B-cell survival signals. This strategy simultaneously targets two key processes: removal of pathogenic CD20-positive B cells and inhibition of compensatory survival signals that promote B-cell repopulation. After rituximab-induced B-cell depletion, residual stromal and immune cells in the synovial microenvironment may upregulate B-cell activating factor (BAFF), creating a survival niche for residual or newly repopulating autoreactive B cells. Increased BAFF signaling may contribute to premature pathogenic B-cell re-expansion, relapse, or secondary non-response. Combining rituximab with an anti-BAFF agent such as belimumab may therefore attenuate compensatory BAFF-mediated survival signaling and support deeper or more durable suppression of pathogenic B-cell activity in selected patients. This approach remains an emerging strategy and requires further validation in adequately powered RA-specific clinical studies. (*Note: This original schematic was created by the authors using BioRender.com.*)

Current research is increasingly focusing on overcoming primary and secondary non-responsiveness caused by anti-CD20 therapy through reasonable combination therapy. B-cell depletion may induce compensatory survival signaling in the synovium: residual stromal and immune cells can upregulate B-cell activating factor (BAFF), supporting survival and re-expansion of autoreactive B-cells (Fig. 5A) [153]. This BAFF-mediated compensatory pathway may contribute to pathogenic B-cell repopulation and early disease recurrence (Fig. 5B). Combining rituximab with the anti-BAFF agent belimumab may attenuate these survival signals. Early clinical evidence suggests that dual B-cell/BAFF pathway inhibition may produce deeper or more durable suppression of disease activity than rituximab alone in selected patients [154]. Other combinations, including rituximab with the JAK inhibitor tofacitinib or downstream modulation of T-cell co-stimulation with abatacept, may also help interrupt pathogenic T–B cell interactions [154, 155].

#### Individualized dosing regimens

Pharmacokinetic monitoring has shown that maintaining the concentration of rituximab above 2 µg/mL in the first three months after infusion may be associated with better clinical efficacy [156]. These results support the further exploration of individualized dosing regimens guided by therapeutic drug monitoring (TDM), rather than relying entirely on empirical retreatment regimens. Compared with fixed retreatment every 6 months, retreatment based on disease activity guidance can reduce the risk of cumulative drug exposure and long-term immunosuppression while maintaining disease control [157]. The REDO trial further showed that in RA patients with stable clinical response, an ultra-low dose maintenance therapy with a dose of 200-500 mg once every 6 months (equivalent to an annual cumulative dose of 400-1000 mg) can maintain the disease activity at a low level for up to 4.5 years without accelerating the joint structure damage [158]. These results suggest that reduced maintenance therapy may be a feasible strategy for patients with stable clinical responses after selection.

From an economic perspective, ultra-low-dose treatment regimens can reduce the cost of drug procurement according to the local drug pricing, dosing regimens, and reimbursement policies.Policy adjustments may further promote their implementation. In China, the domestic rituximab biosimilar HLX01 has been included in the National Reimbursement Drug List (NRDL), reducing the out-of-pocket expenses of patients (Table 4), which provides a policy framework for the wider use of biosimilars and individualized treatment regimens [159, 160].

### Technical advances and translational prospects of newer anti-CD20 antibody platforms

**Fig. 6:**
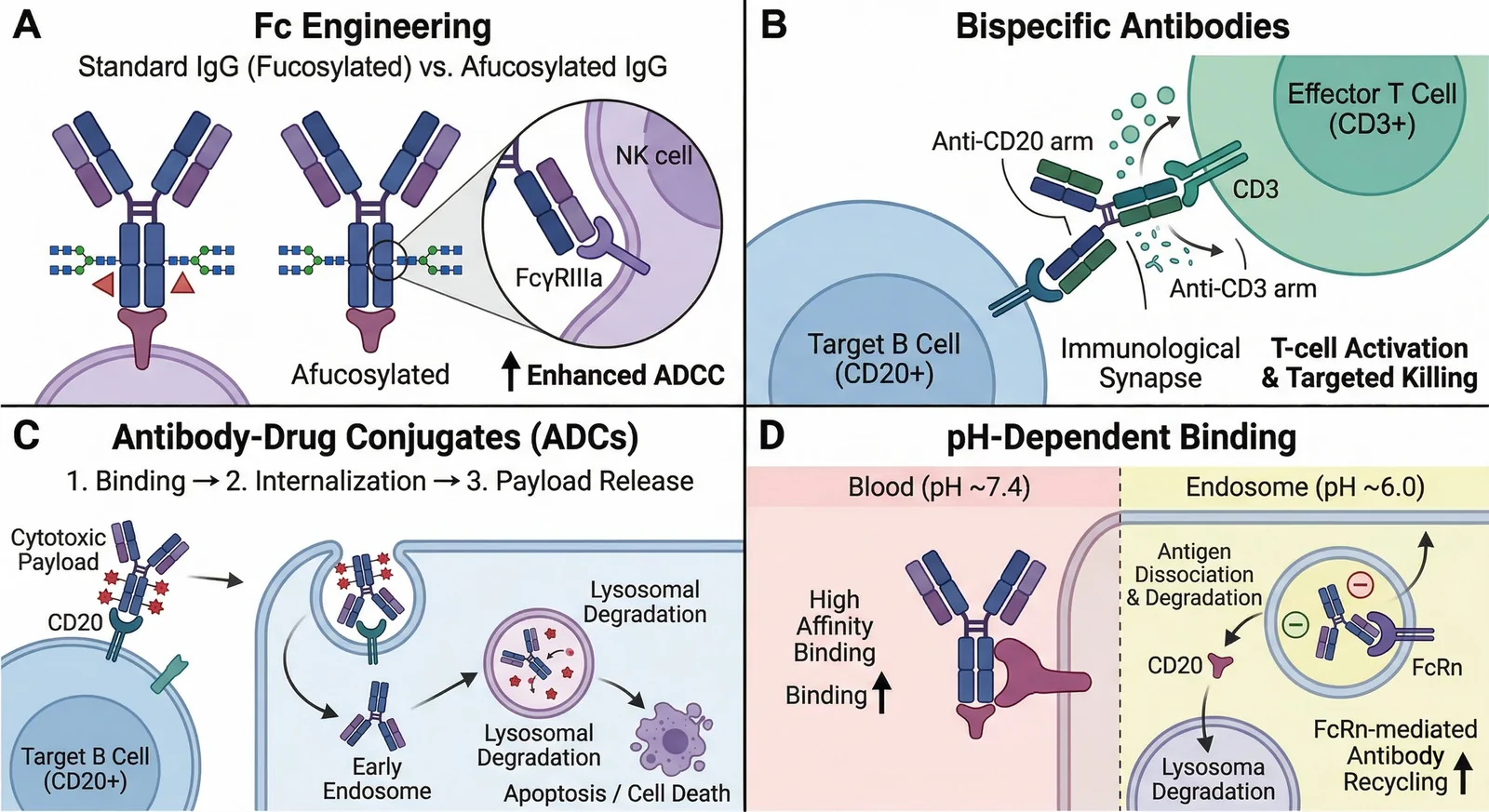
Engineering strategies for newer-generation anti-CD20 monoclonal antibodies. The schematic summarizes four antibody-engineering strategies that may enhance CD20-targeted B-cell depletion, while also highlighting why their translation into RA requires caution. (**A**) Fc engineering: Fc defucosylation can increase affinity for FcγRIIIa on natural killer cells and enhance antibody-dependent cellular cytotoxicity (ADCC), as illustrated by glycoengineered antibodies such as obinutuzumab. (**B**) Bispecific antibodies: CD20×CD3 bispecific antibodies can simultaneously bind CD20 on B cells and CD3 on T cells, facilitating immune synapse formation and T-cell-mediated killing of target B cells. (**C**) Antibody–drug conjugates (ADCs): ADCs combine CD20-targeted binding with intracellular delivery of cytotoxic payloads after internalization and lysosomal processing, thereby inducing target-cell apoptosis. (D) pH-dependent binding and antibody recycling: pH-dependent antibody designs bind CD20 with high affinity at physiological pH but dissociate in acidic endosomal compartments. This allows antigen degradation while enabling antibody recycling through the neonatal Fc receptor (FcRn), potentially prolonging antibody half-life. Although these platforms may improve potency or pharmacokinetics, their use in RA remains largely preclinical or conceptual because chronic autoimmune disease requires a much narrower long-term safety margin than oncology. (*Note: This original schematic was created by the authors using BioRender.com*.)

The implementation of precision-oriented strategies partly depends on the continued evolution of anti-CD20 antibody-engineering platforms. Newer antibody platforms may support future treatment optimization through directed structural modification (Fig. 6). Fc defucosylation can enhance FcγRIIIa binding and increase ADCC activity, as illustrated by obinutuzumab [161]. CD20×CD3 bispecific antibodies can simultaneously engage CD20 on B cells and CD3 on T cells, thereby bridging pathogenic B-cells with effector T-cells and promoting targeted cytotoxicity [162]. Antibody drug conjugates (ADCs) can deliver cytotoxic payloads directly into B-cells after target binding and internalization. pH-dependent antibody designs may prolong antibody half-life by allowing antigen dissociation in acidic endosomal compartments and recycling through the neonatal Fc receptor (FcRn) [159, 163].

However, most of these platforms remain preclinical or early transformation stage in autoimmune disease. Its limitations are consistent with the issues discussed above: rheumatoid arthritis (RA) requires a higher long-term safety threshold than oncology, and excessive immune-cell depletion may increase the risk of immunosuppression, cytokine release syndrome (CRS), and other adverse events. Therefore, in the future, the anti-CD20 platform research in RA should prioritize target specificity, safety monitoring, and treatment accessibility, rather than simply pursuing wider non-specific B-cell depletion. Such strategies are expected to achieve more individualized, lasting, and low-toxicity disease control.

## Conclusions

### Clinical positioning and practical framework for anti-CD20 therapy in RA

The development trajectory of anti-CD20 therapy in RA reflects the transformation of the treatment concept from “extensive immunosuppression” to “precise immunomodulation”. Through the summary of the past 20 years of research and clinical experience, we can find a “next-generation paradox” worthy of attention: antibody engineering technology has promoted the newer generation anti-CD20 antibodies to show stronger B-cell depletion effect and clinical activity in hematological malignancies. However, when it is applied to chronic autoimmune diseases that need long-term treatment, this effect brings the unacceptable risk of opportunistic infections. The limited progress of orelizumab and obinutuzumab in the treatment of RA is an example of this paradox, suggesting that the key to the treatment of autoimmune diseases may not be simply depleted as many pathogenic immune cells as possible but rather achieving long-term balance and sustainable regulation of the immune system.

Therefore, although rituximab has a moderate immunogenicity risk owing to its chimeric structure, it remains plays an important role in modern RA treatment. Currently, targeted drugs such as JAK inhibitors and IL-6 receptor inhibitors have become effective alternatives for RA, because of their rapid onset and strong anti-inflammatory effects. However, for strictly selected patients (especially those with high seropositivity (RF/ ACPA positivity) or those with contraindications for TNF inhibitors or JAK inhibitors, such as demyelinating disease or a history of malignancy), B-cell depletion therapy still has important clinical value.

Notably, the introduction of rituximab biosimilars has expanded the potential clinical and economic value of this strategy. Combined with the ultra-low-dose maintenance treatment strategy supported by the REDO trial, rituximab and its biosimilars may have a better cost-effectiveness ratio in some medical systems than alternative biological agents. The specific effects depend on the drug pricing and reimbursement policies in various regions. This economic advantage makes anti-CD20 therapy not only a salvage treatment strategy, but also an effective way to improve the accessibility of biological agents in resource-limited areas.

Based on the available evidence, we have summarized a set of practical clinical frameworks to assist in the evaluation of the rational application of anti-CD20 therapy in RA, as shown below:

1. Patient selection: Rituximab can be prioritized for seropositive patients with high titers of RF or ACPA antibody, especially when TNF inhibitors or JAK inhibitors are contraindicated or unsuitable.
2. Induction therapy and pharmacoeconomics: When rituximab biosimilars are available and supported by the local regulatory and reimbursement systems, they should be prioritized to use.
3. Maintenance treatment optimization: In patients who achieve sustained low disease activity or clinical remission, dose-reduction or ultra-low-dose maintenance regimens may be considered under close clinical monitoring.
4. Secondary non-response management: In patients with secondary non-response, clinicians should evaluate potential mechanisms such as anti-drug antibodies, B-cell repopulation, or BAFF-mediated compensatory survival signals before considering alternative B-cell-directed or non-B-cell-directed therapies.

The framework does not replace the existing treatment guidelines, but aims to integrate the considerations of efficacy, safety and economics, and provide a reference for the individualized application of anti-CD20 therapy in various medical settings.

### Future research directions

Although the clinical practice framework provides guidance for the rational application of anti-CD20 therapy in RA, the limitations of existing strategies must be overcome to further improve the clinical value of anti-CD20 therapy. The future research direction of RA treatment needs to shift from the “simply pursuing more effective B-cell depletion antibodies” to “a more clinically feasible and precise optimization path”, specifically including the following:

- Develop accessible biomarkers based on routine blood detection to improve patient selection efficiency and predict treatment response.
- Establish individualized dosing regimens (especially ultra-low-dose maintenance strategies) oriented by treatment goals, and verify their long-term safety and effectiveness in different patient subgroups in multicenter prospective studies.
- Conduct the large-scale prospective clinical trials of B-cell-BAFF pathway combination strategies.
- Conduct the pharmacoeconomic research on regional adaptability in combination with the allocation of medical resources, drug price system and reimbursement policy in different regions.

These research directions may help to promote the transformation of anti-CD20 therapy from the empirical therapy to precise individualized strategy, so as to maintain its specific clinical value in the fiercely competitive field of RA biotherapy.

### Limitations of this review

It should be noted that clinical positioning, practical frameworks, and future research directions are all based on existing evidence, and this review has methodological limitations.

First, this article adopted a narrative review design instead of informal systematic evaluation or quantitative meta-analysis. Therefore, this study did not adopt the PRISMA process, formal bias risk assessment and the quantitative evidence summary. Because of the heterogeneity in population characteristics, background treatment, retreatment strategies, endpoints, and follow-up durations, the direct comparison and quantitative integration of cross-research results are limited to some extent.Therefore, the conclusion of this review should be interpreted in the methodological background of narrative evidence integration.

Second, the evidence base for newer-generation anti-CD20 antibodies in RA is still far weaker than that in hematological malignancies or multiple sclerosis. Therefore, several discussions on ocrelizumab, ofatumumab, obinutuzumab, bispecific antibodies, antibody-drug conjugates, and other emerging therapeutic platforms are mainly based on mechanism extrapolation rather than high-quality clinical evidence of RA, and should not be regarded as conclusive proof of efficacy or safety for RA.

Third, pharmacoeconomics conclusions are highly regionally dependent. There are significant differences in drug procurement costs, biosimilars penetration rates, reimbursement policies, and medical service systems among countries and regions. The real-world prescription data and cost-benefit researches cited in this article can provide a reference for the competitive pattern in the field of RA treatment. However, these studies cannot substitute for region-specific RA market-share analyses, prescription-claims data studies, or localized cost-effectiveness evaluation. Therefore, the economic judgement presented in this article should not be directly extrapolated to all clinical decision-making scenarios, but should be interpreted in combination with the local medical practice background and policy environments.

Fourth, the effectiveness and long-term safety of biomarker-guided patient selection,, synovial transcriptome profiling, individualized ultra-low-dose maintenance, and B-cell-BAFF dual-targeting strategies remain insufficiently validated for routine RA clinical practice, and need to be further verified through large-scale prospective clinical studies.

Taken together, anti-CD20 therapy retains a selective but clinically important role in contemporary RA management, with its value concentrated in appropriately selected patient populations rather than uniformly across all RA phenotypes. Rituximab remains an established anti-CD20 option in RA and is supported by substantial RA-specific clinical evidence, whereas the clinical relevance of newer-generation antibodies and emerging engineering platforms remains uncertain. Biosimilars and individualized retreatment strategies may improve treatment accessibility and real-world feasibility, but their implementation should be considered within local reimbursement settings and accompanied by appropriate monitoring of immunoglobulin levels, infections, and B-cell reconstitution. Future advances will require prospective validation of clinically accessible biomarkers, safer and more individualized retreatment strategies, RA-specific evaluation of combination approaches, and context-specific pharmacoeconomic analyses. Ultimately, the long-term value of anti-CD20 therapy is likely to depend less on maximizing B-cell depletion intensity alone than on achieving durable disease control while preserving immune competence, maintaining treatment accessibility and ensuring long-term clinical feasibility.

## Data Availability

No datasets were generated or analysed during the current study. All information discussed in this narrative review was derived from previously published sources cited in the manuscript.

## Abbreviations

ACPA: Anti-citrullinated protein antibody
ACR: American College of Rheumatology
ACR20/50/70: American College of Rheumatology 20%, 50%, and 70% response criteria
ADA: Anti-drug antibody
ADC: Antibody–drug conjugate
ADCC: Antibody-dependent cellular cytotoxicity
ADCP: Antibody-dependent cellular phagocytosis
APC: Antigen-presenting cell
APRIL: A proliferation-inducing ligand
BAFF: B-cell activating factor
BCR: B-cell receptor
bDMARD: Biological disease-modifying antirheumatic drug
CDC: Complement-dependent cytotoxicity
CLL: Chronic lymphocytic leukemia
CRS: Cytokine release syndrome
csDMARD: Conventional synthetic disease-modifying antirheumatic drug
DAS28: Disease Activity Score in 28 joints
DCD: Direct cell death
ELS: Ectopic lymphoid structure
EMA: European Medicines Agency
FDA: US Food and Drug Administration
FcRn: Neonatal Fc receptor
HBV: Hepatitis B virus
HEV: High endothelial venule
IVIG: Intravenous immunoglobulin
JAK: Janus kinase
MAC: Membrane attack complex
MS: Multiple sclerosis
MTX: Methotrexate
mTSS: Modified total Sharp score
NK: Natural killer
NMPA: National Medical Products Administration
NMSC: Nonmelanoma skin cancer
NRDL: National Reimbursement Drug List
PBM: Pharmacy benefit manager
PML: Progressive multifocal leukoencephalopathy
PRISMA: Preferred Reporting Items for Systematic Reviews and Meta-Analyses
RA: Rheumatoid arthritis
RF: Rheumatoid factor
RTX: Rituximab
scRNA-seq: Single-cell RNA sequencing
TDM: Therapeutic drug monitoring
TNFi: Tumor necrosis factor inhibitor
Tph: Peripheral helper T cell
tsDMARD: Targeted synthetic disease-modifying antirheumatic drug
